# Potential of Reusing 3D Printed Concrete (3DPC) Fine Recycled Aggregates as a Strategy towards Decreasing Cement Content in 3DPC

**DOI:** 10.3390/ma17112580

**Published:** 2024-05-27

**Authors:** Szymon Skibicki, Karol Federowicz, Marcin Hoffmann, Mehdi Chougan, Daniel Sibera, Krzysztof Cendrowski, Mateusz Techman, João Nuno Pacheco, Maxime Liard, Pawel Sikora

**Affiliations:** 1 Faculty of Civil and Environmental Engineering, West Pomeranian University of Technology in Szczecin, 70-310 Szczecin, Poland; karol.federowicz@zut.edu.pl (K.F.); daniel.sibera@zut.edu.pl (D.S.); kcendrowski@zut.edu.pl (K.C.); mateusz.techman@zut.edu.pl (M.T.); 2 Faculty of Mechanical Engineering and Mechatronics, West Pomeranian University of Technology in Szczecin, 70-310 Szczecin, Poland; marcin.hoffmann@zut.edu.pl; 3 Department of Civil and Environmental Engineering, Brunel University London, Uxbridge UB8 3PH, UK; 4 CERIS/c5Lab—Sustainable Construction Materials Association, Edifício Central Park, Rua Central Park 6, 2795-242 Linda-a-Velha, Portugal; jpacheco@c5lab.pt; 5 Sika Technology AG—Central Research, Tüffenwies 16, 8064 Zurich, Switzerland

**Keywords:** 3D concrete printing, 3DPC, recycled aggregate, additive manufacturing, rheology, green strength, concrete, waste aggregate

## Abstract

This paper explores the new potential strategy of using fine recycled aggregates (fRA) derived from waste 3D printed concrete (3DPC) as a substitute for cement in additive manufacturing. This study hypothesizes that fRA can optimize mixture design, reduce cement content, and contribute to sustainable construction practices. Experimental programs were conducted to evaluate the fresh and hardened properties, printability window, and buildability of 3DPC mixes containing fRA. Mixes with replacement rates of cement with fRA by 10 vol%, 20 vol%, 30 vol%, 40 vol%, and 50 vol% were produced. A comprehensive experimental protocol consisting of rheological studies (static and dynamic yield stress), dynamic elastic modulus determination (first 24 h of hydration), flexural and compressive strengths (2 d and 28 d), and an open porosity test was performed. The obtained results were verified by printing tests. In addition, an economic and environmental life cycle assessment (LCA) of the mixes was performed. The results indicate that up to 50 vol% cement replacement with fRA is feasible, albeit with some technical drawbacks. While fRA incorporation enhances sustainability by reducing CO_2_ emissions and material costs, it adversely affects the printability window, green strength, setting time, and mechanical properties, particularly in the initial curing stages. Therefore, with higher replacement rates (above 20 vol%), potential optimization efforts are needed to mitigate drawbacks such as reduced green strength and buildability. Notably, replacement rates of up to 20 vol% can be successfully used without compromising the overall material properties or altering the mixture design. The LCA analysis shows that reducing the cement content and increasing the fRA addition results in a significant reduction in mix cost (up to 24%) and a substantial decrease in equivalent CO_2_ emissions (up to 48%). In conclusion, this study underscores the potential of fRA as a sustainable alternative to cement in 3D printed concrete.

## 1. Introduction

The construction industry faces significant challenges in maintaining sustainability within today’s environmental context. This is primarily due to the cement industry grappling with the contradicting factors of meeting the substantial demand for raw clinker production and minimizing the industry’s CO_2_ emissions [1].

The rapid urbanization and post-war demolition in Europe have resulted in significant amounts of construction and demolition waste (CDW), attracting academic attention due to its potential for secondary use [2]. The movements mentioned above contribute significantly to total waste generation. In the European Union (EU), CDW accounts for over 36% of all waste produced [3]. The EU has established ambitious objectives for upcycling CDW in response to this challenge. Leveraging CDW as a secondary raw material offers dual environmental advantages, such as decreasing the demand for mineral natural resources and diminishing the waste sent to landfills.

Fine recycled aggregate (fRA) utilization practices commonly entail low-value applications like backfilling, relegating its potential to landfills, or indefinite stockpiling at CDW facilities. This highlights the importance of setting specific goals for fRA under the European Union’s Circular Economy Action Plan [3]. To advance the market reach of fRA, a shift towards more valuable applications is necessary. This can be facilitated through innovative methods like additive concrete manufacturing, which can drive a paradigm shift. This strategic reorientation aligns with circularity principles and emphasizes the importance of using technological advancements to promote sustainable resource utilization in the construction industry.

This paper concerns the applicability of fine recycled aggregates (fRA) produced from 3D printed concrete waste (in practical terms, ultra-high-performance concrete (UHPC) waste). Research has demonstrated that recycled aggregates produced from ultra-high-performance concrete (UHPC) tend to be better suited for use in concrete than recycled aggregates produced from lower-quality waste [4]. This is partly due to the higher cement content of UHPC, often around 1100 kg/m^3^, which is more than three times that of the cement content of conventional concrete [5]. However, using recycled aggregates in concrete can adversely affect its fresh and hardened properties [6].

With the advent of concrete 3D printing, specific criteria such as extrudability, buildability, and setting time must be met through the mixed formulation design of 3D printed concrete (3DPC). As a result, a substantial quantity of binder, primarily cement, is needed, with a quantity exceeding 700 kg/m^3^, which is more than two times that of conventional concrete [7,8]. Pacheco et al. [9] conducted a literature survey that concluded that most 3DPC mixes contain binder contents exceeding 500 kg/m^3^. In addition, aggregate sizes greater than 4 mm are typically excluded from 3DPC mix designs, with a maximum aggregate size of 2 mm being recommended. This requirement is due to pump limitations and the need for narrow cross-section layers, resulting in a considerable demand for river sand in 3D printing. This poses a severe risk to natural resources, due to the high ratio of fine aggregates. In conjunction with the increasing real-world developments in concrete 3D printing, the above facts have demonstrated that recycled aggregates obtained from 3DPC waste have immense potential to serve as a secondary material source after recycling.

Currently, there is a shortage of knowledge regarding the use of fRA in 3D printing. Employing recycled aggregates as a substitute for natural aggregates is considered a viable solution to tackle the issue of CDW accumulation and minimize the extraction of natural resources [10,11]. For instance, Ding et al. [12] studied the impact of incorporating fine recycled aggregates on the mechanical performance of 3D printed concrete. Their results indicated a slight decrease in the compressive and flexural strengths of the 3D printed samples that contained fine recycled aggregates compared to those that used natural sand. Moreover, the compressive, tensile splitting, and flexural strengths of the recycled sand 3D printed concrete were significantly anisotropic. As previously stated, while there may be some slight decay in performance, research has shown that 3D printed concrete made using recycled aggregates still performs satisfactorily [12,13,14,15]. Additionally, several proposed methods have been made for improving the properties of these aggregates, including carbonation modification, which has been proven effective at enhancing the recycled fine aggregates and aiding in carbon dioxide sequestration [11].

This research explores the potential use of recycled fine aggregates from 3DPC waste in additive manufacturing as a substitute for cement. While one paper [16] has reported incorporating recycled fine powder waste as a cement substitute, it required grinding the CDW down to less than 75 microns. To the best of our knowledge, no study has yet examined the feasibility of using fRA derived from recycled 3DPC waste as a cement substitute in 3DCP without adding extra mixing water and applying extreme energy-consuming grinding processes.

Although some of the characteristics of fRA, such as high water absorption and rough texture, are undesirable for conventional concrete, if a proper mix design is carried out, they are advantages for a 3D printed concrete mix design, since they may be used to tailor the rheological properties of a 3D printed mix (ensure proper buildability and extrudability), allowing decreases in cement content. To date, there is limited knowledge on the application of fRA for 3D printing applications; however, some research groups have confirmed their satisfactory performance in 3D printed concrete [12,14,17].

Based on the abovementioned literature and scarce information on the design of 3DPC with fRA, a preliminary study was conducted to optimize the mix design by including fRA produced from the recycling of 3DPC. The following hypotheses were studied in this paper:

- The rougher texture and the much higher water absorption of an fRA in comparison to natural fine aggregates imply that an extrudable mix increases its strength earlier, increasing the buildability as time passes (but decreasing extrudability);
- If a proper balance is reached, optimizing the mixture design to achieve lower cement content by introducing fRA in the mix is possible.

These hypotheses were the basis for an experimental program in which the following occurred:

- Starting with a reference mix, mortars for 3DPC were developed by replacing the cement with fRA at different replacement ratios;
- The fresh-state hardened mechanical properties and the extrudability and buildability of the 3DPC mixes tested were studied;
- To better understand the influence of the fRA on the properties of 3DPC, the superplasticizer dosage remained fixed for all mixes;
- The 3D printing process of the testing columns was performed in the same printing conditions (fixed printing parameters, including speed and methodology);
- A comparative life cycle assessment (LCA) was used to quantify the environmental and economic benefits of replacing cement with fRA.

As a first approximation, it was assumed that no changes to the water content of the mix were needed. This is because cement replacement (smaller particle size and higher surface area) is compensated by rough surface of fRA, which has high water absorption rates. This hypothesis was compared with a workability window that was defined based on slump flow testing.

The assumptions and experimental design are part of a comprehensive research program to find alternatives for using fRA in 3DPC. The present study aims to simplify a mix design procedure that maximizes the environmental benefits of this replacement (since cement is replaced with fRA without additional changes to the mix design) at a technical cost (presumed loss of open time, workability, and extrudability). Other experiments are ongoing and aim to maximize the fresh- and hardened-state technical performance.

## 2. Materials and Methods

### 2.1. Materials

**Figure 1:**
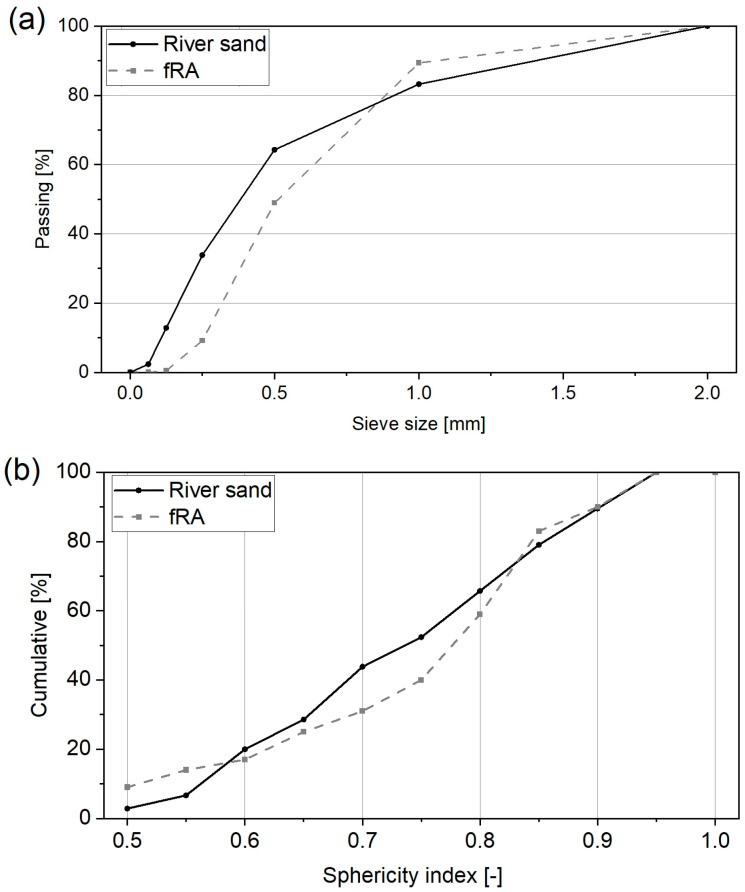
Particle size distribution (**a**) and particle sphericity index (**b**) of natural and recycled aggregates.

**Figure 2:**
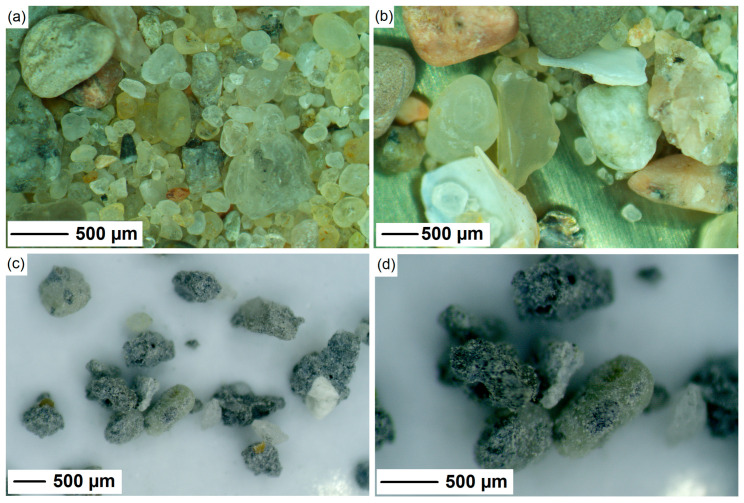
Optical microscope image of NA (**a**,**b**) and fRA produced from waste 3DPC (**c**,**d**).

The binder phase of the 3DPC mix studied in this paper comprises CEM I 42.5 R, fly ash, and silica fume. In the control mix (R0), the ratio of dry components was fixed by mass at 70:20:10 (cement:fly ash:silica fume). To ensure proper rheological properties, Sika^®^ Viscocrete^®^ P-111 superplasticizer (SP) (Conroe, TX, USA) was used, and the amount of SP was fixed to solely evaluate the effects of fRA presence in the mixture. Natural river sand (NA), with particle size below 2 mm, was obtained from SKSM (Szczecin, Poland). The source material used to produce the recycled fine aggregates (fRA) was waste 3DPC from previous laboratory experiments at the West Pomeranian University of Technology in Szczecin. The waste material was, on average, one year old, and its compressive strength was typically above 50 MPa. Exemplary compositions of the mixes can be found in [7,8]. A single jaw crusher (LAB-08-240, EKO-LAB, Kraków, Poland) was used to process the concrete waste into recycled aggregates, which were sieved through a 2-mm sieve. The particle size distribution of the aggregates is shown in Figure 1a. The natural aggregate (river sand) had finer particles, typical to deposits available in the region of origin, while the distribution of fRA was in line with the typical grading of conventional sands. Concerning shape, even though elongation and flakiness indices cannot be evaluated for fine recycled aggregates through optical microscopy (Figure 2), the authors calculated the sphericity index (Figure 1b) of several particles (>45 particles) and found that the fRA had an angular shape (several particles with a sphericity index of ≤0.55). Such particles are visible in Figure 2a,b, while the natural aggregates tended to be more spherical (Figure 2a,b). This was already expected. Since the natural aggregate was river sand, sphericity was to be expected, while the fRA were produced by a single jaw crushing process, which tends to result in elongated (flaky) shapes [18], such as those shown in Figure 2.

The properties of the aggregates are summarized in Table 1. As expected, the fRA exhibited lower density and higher water absorption than the natural aggregate. However, the density was found to be relatively high (2400 kg/m^3^), while the water absorption was relatively low (6.4%) compared to the typical values obtained for fine recycled aggregates (fRA) from construction and demolition waste (CDW). For reference, the average values from the literature suggest a density of 2295 kg/m^3^ and water absorption of 8.4% based on 38 sources collected in [9]. This is attributed to the high quality (including high strength, low water-to-binder ratio, and high cement content [9]) of the waste used to produce the fRA.

**Figure 3:**
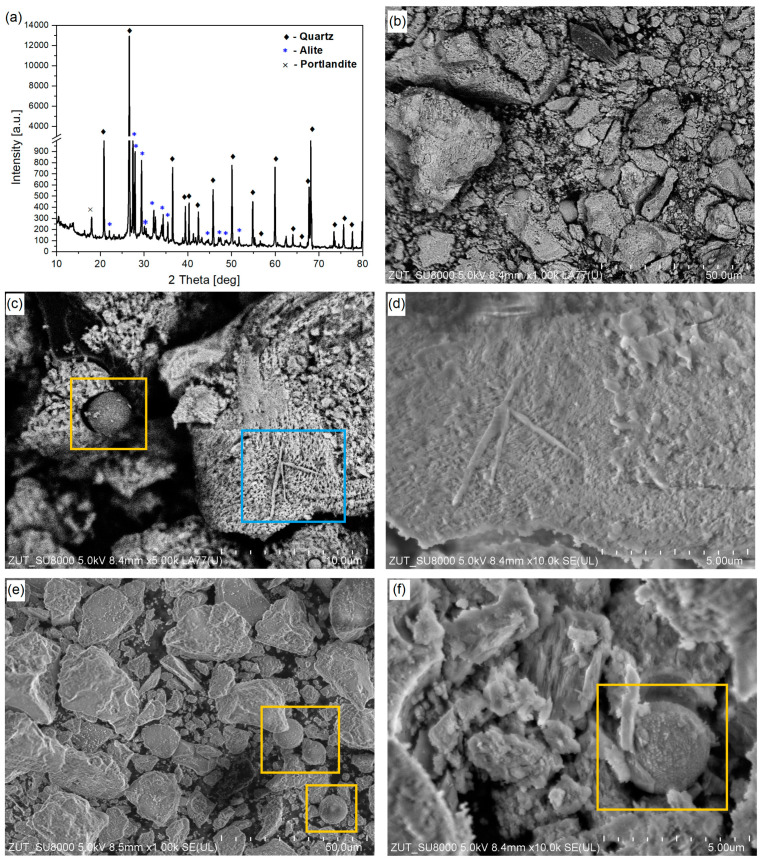
X-ray diffraction pattern (**a**) and SEM images (**b**–**f**) of fRA. Orange boxes indicate fly ash particles, while the blue box indicates Portlandite.

The microscopy analysis used to plot Figure 1b is presented in Figure 2 and further discussed here. The differences in the morphology of the aggregates are easily observed, as follows: the river sand aggregates are mostly siliceous-based aggregates with round particles, while the fRA are elongated and slightly porous mortar attached to virgin aggregate particles. Figure 2 shows not only the differences in shape, but also in roughness, as follows: due to the nature of the materials, most natural aggregates have a smooth surface, while the fRA are rough (this has consequences for the fresh-state behavior of the mortars, as discussed in the Introduction). Additional scanning electron microscope (SEM) analysis of the fRA particles confirmed their elongated and needle-shaped particle morphology (Figure 3b), along with visible particles of non-reacted fly ash (Figure 3c,e,f). Moreover, tracks of Portlandite were observed (Figure 3c,d), which was confirmed by X-ray diffraction (XRD) analysis (Figure 3a). The phase composition was determined using the X-ray diffraction method, Cu Kα radiation (λCu Kα = 0.1540 nm), on an Empyrean (Panalytical, Malvern, UK). The identification of the crystalline phases was performed using HighScore Plus 3.0d (3.0.4, produced by PANalytical B.V., Almelo, The Netherlands) and the ICDD PDF-4+ 2015 database (access date: 1 March 2024). The XRD pattern shows that the main mineral composition of the 3DPC fRA was quartz, which came from the original (natural) aggregates. At the same time, unhydrated cement particles were also observed, as indicated by the peaks of alite (C_3_S). Moreover, the presence of Portlandite was reported. This can be attributed to the relatively low w/b and high binder content of the 3DPC. As reported in previous studies [19,20], such a mineral composition of recycled fine concrete powders can potentially exert pozzolanic and filler effects in the cement matrix. Therefore, an additional assessment of the finest fRA (<0.125 mm) fraction was performed.

A strength activity index (SAI) test conforming to ASTM C311-11 was performed for initial screening. Although it is widely agreed upon in the literature that this test is not wholly accurate for materials of low reactivity, it can provide some general insight into the potential reactivity [21] of recycled fines. According to the abovementioned standard, the cement mortar with the 20% cement replacement with the finest fractions of fRA (<0.125 mm) was produced, and its compressive strength was compared with the control mortar containing pure cement after 7 and 28 days of curing. The SAI index (set by ASTM C618) should equal at least 75% to consider the material as pozzolanic. In this case, the mortar containing fRA reached 76% and 75% of reference compressive strength (plain cement), thus at the threshold. Therefore, an additional testing method suitable for pre-screening low-reactive materials was carried out (the modified R^3^ test proposed in [22,23]). The heat of the hydration of the system containing the fRA fraction < 0.125 mm was evaluated using an isothermal calorimeter showing the reactivity of the filler as 149 J/g_filler_, which indicated that the finest fraction of fRA exhibited minimal reactivity.

### 2.2. Mixture Design and Mixing Methodology

A set of 3DPC mixes was produced by replacing cement with fRA up to 50 vol%, with gradual replacement increments of 10 vol% (see Table 2). The replacement of cement with fRA resulted in very relevant cement decreases from 560 kg/m^3^ (R0) to 280 kg/m^3^ (R50), accompanied by an 81% increase in the aggregate-to-binder (a/b) ratio. At the same time, the water-to-binder (w/b) ratio increased from 0.28 (R0) to 0.43 (R0). Therefore, in the proposed strategy, fRA was used without adding water to compensate for the roughness and water absorption of the fRA.

The mix preparation procedure was as follows: (1) the dry binder components were mixed to receive a uniform powder (45 s ± 5 s); (2) water with superplasticizer (SP) was added to the dry binder components, then mixed for 3 min to obtain a homogeneous paste; and (3) natural and recycled aggregates (fRA) were added to the paste and mixed for 4 min to achieve an entirely homogeneous mixture. All mixes were prepared under laboratory conditions at a temperature of 20 °C ± 2 °C and relative humidity (RH) of 50% ± 10%.

### 2.3. Rheological Properties Measurement

The rheological properties of the 3DPC were determined using a modular compact rheometer MCR 72 (Anton Paar). A testing set of vane geometry and a customized ribbed cup with a volume of v = 120 cm^3^ (to exclude potential wall-slip effect) were used. The static yield stress (SYS) was determined at a shear rate of 0.1 s^−1^ in the function of time, up to 45 min, by adding the water to the dry components (T_0_), following the methodology presented by Chen et al. [9]. Similarly, dynamic yield stress (DYS) was determined by applying a shear rate from 100 s^−1^ to 0.1 s^−1^, with 31 points distributed evenly along a logarithmic scale, for a total of 300 s. The tests were preceded by pre-shearing at 100 s^−1^ for 60 s. The DYS test was performed after 15 min from T_0_. The consistency of the developed material was measured using the flow table test, conforming EN 1015-3 [24] after 15, 30, and 60 min from T_0_.

### 2.4. Green Strength Evaluation

The green strength of the 3DPC was assessed by a uniaxial unconfined compressive test. The tests were performed using a special precision press device with a precise LVDT and a force sensor (up to 500 N, HBM C9C 0.5 kN, Darmstadt, Germany) coupled to a HBM MGC Plus AB22A bridge (HBM, Darmstadt, Germany). The device and testing methodology were described comprehensively in [13,25]. The tests were conducted on the cylindrical specimens (D = 60 mm, H = 120 mm). In addition, the green Young Modulus was evaluated using the procedure presented in [25] based on the linear part of the stress–strain curve between strains $\epsilon_{u}=0.06$ and $\epsilon_{d}=0.04$. For all obtained data, the value of the coefficient of variation (CoV) was calculated. The green strength was evaluated after 30 and 60 min after water contact.

### 2.5. Dynamic Elastic Modulus Evaluation

The dynamic elastic modulus was determined using a Vikasonic apparatus (Schleibinger, Ulm, Germany). The apparatus utilizes a typical Vicat ring, to which two ultrasonic transducers are connected. The equipment sends a pulse every 1.0 s at a frequency of 54 kHz. The results were monitored for an initial 24 h after water–cement contact. The dynamic modulus of elasticity (E_dyn_) was determined based on the velocity (v) and density ρ of prepared mixes using the relation (Equation (1)), as follows:

(1) $$E_{dyn}=\rho \;\cdot \;v^{2}$$

### 2.6. Density and Porosity of Tested Specimens

A hydrostatic scale was used to determine the oven-dry density and open porosity of the specimens based on Archimedes’ principle. The test was performed after 28 d of curing on three cubical specimens of 40 × 40 × 40 mm^3^. The specimens’ underwater and saturated masses were determined in the first stage. Afterward, the specimens were dried in a laboratory oven until a constant mass was reached. From the data, the oven-dry density and open (capillary) water porosity were determined. The details of the experimental technique can be found in [26].

### 2.7. Hardened Mechanical Properties

The flexural and compressive strengths were determined for cast prismatic specimens with a standard size (40 × 40 × 160 mm^3^) after 2 and 28 days of curing. The tests were performed according to EN 1015-11 [27].

### 2.8. Printing Test and Buildability Evaluation

**Figure 4:**
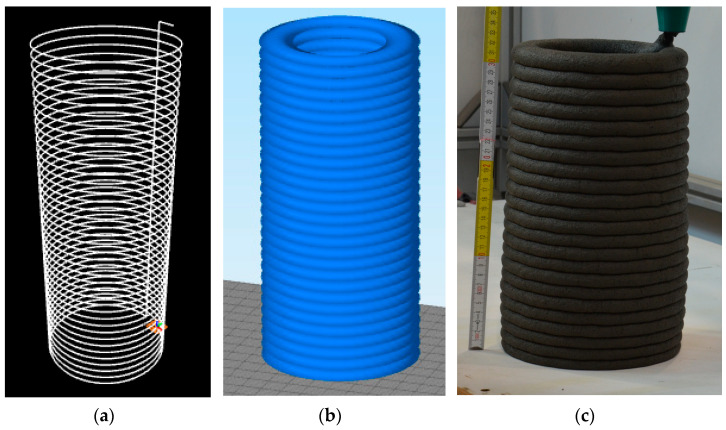
G-Code path (**a**), 3D model of printed column (**b**), and column during printing process (**c**).

The printing was performed with a 3-degrees-of-freedom Cartesian robot attached to a pumping module. The mixture was extruded using a screw head at a flow rate of approximately 1.25 ± 0.10 L/min. The head had a nozzle with an outlet diameter D of 25 mm. This work describes the printing system used in detail [25]. The dimensions of the printed path were as follows: height 14 mm and width 30 mm. The printing test consisted of printing a column with a diameter of 150 mm, a height of 420 mm, and a printing speed of 50 mm/s. A minimum of three columns for each mix were printed. The G-Code path, a 3D model of the designed column, and a photograph of the column during printing are presented in Figure 4.

If the mix’s buildability was insufficient to achieve the mentioned height, the test ended due to structure collapse. In this case, the maximum height of the specimen was measured as the height limit. Additionally, a visual inspection of element quality was performed.

### 2.9. Life Cycle Assessment: Methodology

The LCA aimed to understand the potential economic and environmental benefits (evaluated through the global warming potential defined in EN15804:2012+A2:2019 [28]) of using fRA as a partial replacement for cement. This was achieved by comparing the cost and global warming potential (equivalent CO_2_ emissions) of the different mixes tested in this paper. Due to the conceptual nature of the paper, the data on the cost of raw materials and transport were sourced from reference values in Poland, rather than from a specific case study of 3DPC (which are scarce). The LCA concerned the cost and global warming potential, and the analysis was carried out from the cradle to the grave, covering information modules A1 (raw material production), A2 (transport of raw materials to production site), and A3 (production of the 3DPC mortars), according to EN15804:2012+A2:2019 [28]. Module A3 was disregarded, since (i) it had a minimal impact (well below cut-off rates), as explained in Section 2.9; and (ii) the paper evaluated the technical, environmental, and economic impacts of changing the composition of a reference 3DPC mix. This change had no implications for mixing.

For each raw material and transport method, the global warming potential data were sourced from secondary data from (i) Environmental Product Declarations (EPDs), (ii) the Ecoinvent database [29], and (iii) peer-reviewed publications. Section 3.7 presents the results of the LCA.

## 3. Results

### 3.1. Fresh Properties

**Figure 5:**
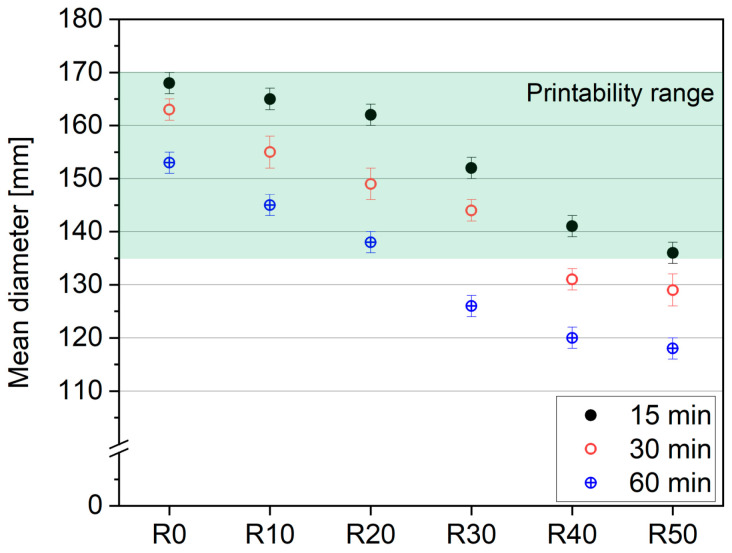
Flow table of 3DPC mixes at various testing times.

Figure 5 illustrates the spreading flow of the 3DPC mixes as a function of time. The flow table test is widely agreed to be a simple method for screening the potential printability range (open time) of the mixture, and a range of 135–170 mm is considered printable [30,31,32,33,34]. A higher flow was reported for specimens with lower replacement rates and mixes R0–R20, which exhibited printability for up to 60 min. The higher replacement rates (from 30 vol%) resulted in a substantial decrement in the mix’s printability; therefore, R40 and R50 were found to be printable for less than 30 min, which is enough when the mixes are produced continuously before being printed. The reduction in workability can be attributed to the increased volume fractions of aggregates resulting from the substitution of cement with the fine recycled aggregate (fRA). There was a gradual decrease in the volume fractions of the paste, composed of cement, fly ash, silica fume, and water, from approximately 50% (R0) to 40% (R50). This decrease led to a corresponding reduction in workability, culminating in the jamming of aggregates at a volume fraction of up to 60% (R50). Additionally, the loss of workability was affected by the increased air entrainment, which accompanied the reduction in paste volume. The increase in the polycarboxylate ether (PCE) content by cement weight (the PCE content was fixed for all mixes, but the cement content decreased with the fRA incorporation) decreased the yield stress of the paste. However, this reduction was insufficient to counterbalance the flowability loss of the mixes fully. The flow was predominantly governed by the aggregate content rather than the fluidity of the paste. This is evident in the samples R40 and R50, where segregation in the flow was observed, highlighting the substantial impact of aggregate content on the overall mix behavior.

**Figure 6:**
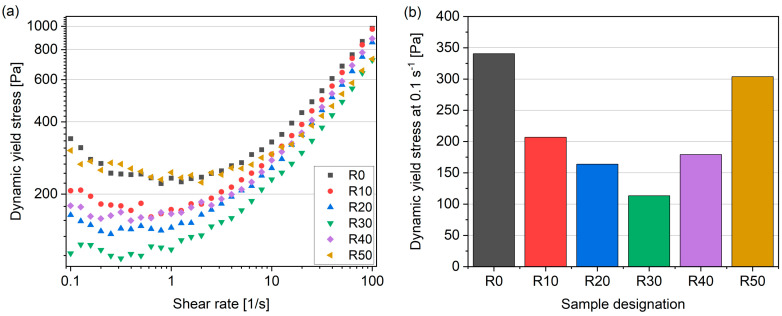
Shear stress as a function of the shear rate (**a**) and dynamic yield stress at 0.1 s^−1^ (**b**) of 3DPC mixes performed after 15 min from T_0_.

Rheological studies were conducted to assess the influence of fine recycled aggregates (fRA) on the fresh-state properties of the mortars. Figure 6a illustrates the variation in shear stress as a function of shear rate for the different mixes. In the R40–R50 samples, notable anomalies were observed, characterized by a decreased plastic viscosity and increased dynamic yield stress measurements (Figure 6). These anomalies are likely due to increased air entrapment in the mixes. The measurements, ranging from 100 to 0.1 s^−1^, reveal an increase in shear stress from 1 to 0.1 s^−1^ for nearly all of the mixes. This increase is attributed to thixotropy, indicating a time-dependent evolution of shear stress at low shear rates. The reference mix (R0) exhibited significantly higher thixotropy than the R20 and R30 mixes, primarily due to its lower relative PCE content (to cement weight). It has also been observed that the decrease in plastic viscosity is mainly attributed to the increased air entrainment accompanying the addition of fRA.

**Figure 7:**
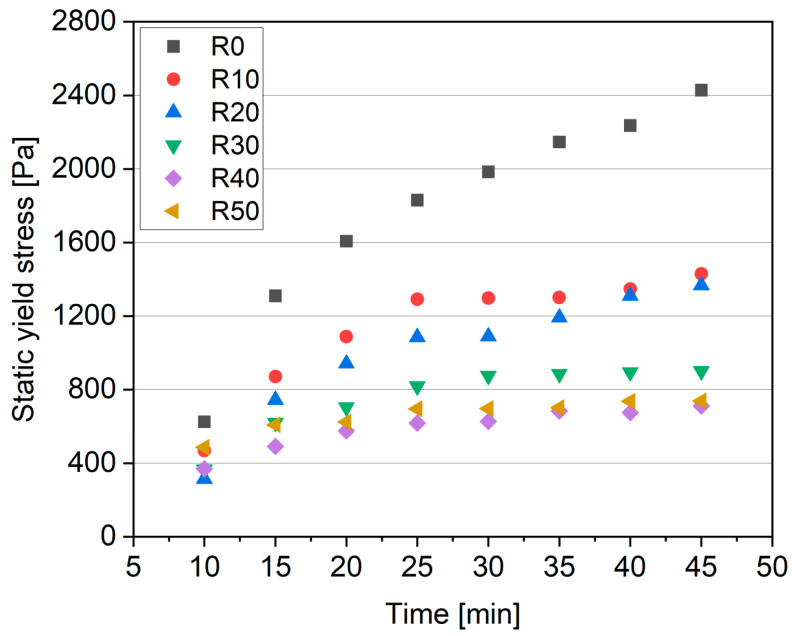
Static yield stress development of 3DPC as a function of time.

A depiction of static yield stress development is illustrated in Figure 7. This evolution provides insights into the material’s ability to support vertical layer deposition (e.g., a measure of buildability). There is a discernible reduction in the static yield stress growth in the samples with an increasing fRA and decreasing cement content. Notably, after 45 min, the static yield stress values for the R30–R50 samples were similar to those observed for the R0 mix at 10 min. This trend can be attributed to the increased concentration of PCE in the mixture containing a higher volume contribution of aggregates (natural + fRA), along with a lower volume content of paste. As the PCE concentration increases with the increase in fRA (and decrease in cement), there is a corresponding decrease in the thixotropic behavior of the mixtures.

### 3.2. Green Strength Evaluation

**Figure 8:**
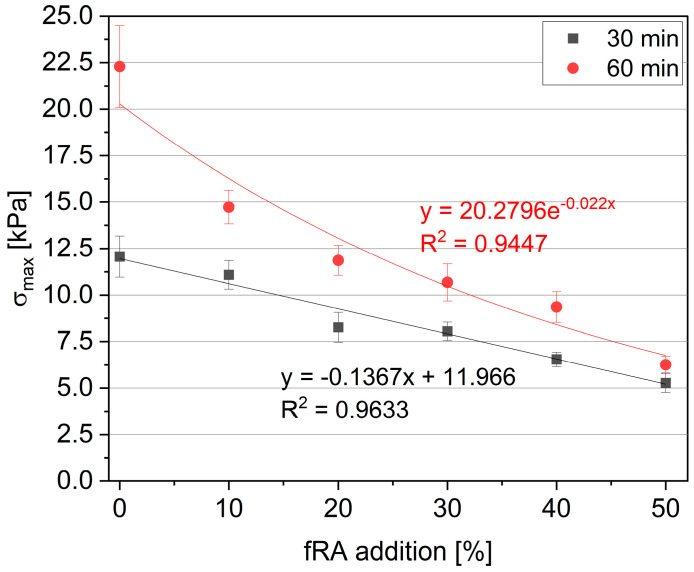
Relationship between fine recycled aggregate (fRA) and maximum “green” strength.

**Figure 9:**
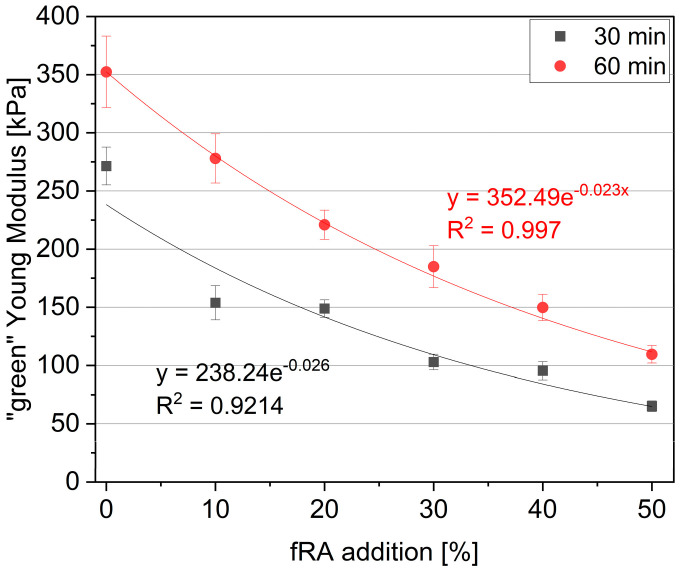
Relationship between the fine recycled aggregate (fRA) and “green” Young’s Modulus.

Figure 8 presents the relationship between the green strength and fRA addition, while Figure 9 presents the relationship between the fine recycled aggregate (fRA) and “green” Young Modulus. The CoV (Coefficient of Variation) values for the green strength and Young’s modulus ranged from 6.16% to 9.70% and 5.05% to 9.75%, respectively. The green properties, including green strength and green Young’s modulus, significantly decreased with the addition of fRA. In addition, both plots compared the values determined after 30 and 60 min of water contact (T_0_). It should be noted that the green strength after 30 min decreased linearly with the increase in fRA content. The green strength reduced to 56.32% (mix R50) compared to R0. After 60 min of water contact, the exponential reduction in green strength was visible. It should be noted that even a 10% fRA addition (R10) resulted in a 33.93% decrement in green strength, while maximum replacement resulted in a 71.93% decrement (R50) when compared to R0. As an outcome, the green strength for all mixes ranged between 5.27 kPa and 12.07 kPa for the test after 30 min and 6.26 kPa and 22.29 kPa for the test after 60 min.

As expected, the green Young’s modulus followed a similar trend as the green strength. However, it should be noted that both Young’s modulus results (after 30 and 60 min) decreased exponentially with increasing fRA content. It should also be noted that a dramatic reduction in Young’s modulus was reported (after 30 min), even for specimens containing 10 vol% and 20 vol% of fRA inclusion (43.33% and 45.16% loss in comparison to R0, respectively). The Young’s modulus decreased to 75.98% and up to 68.92% for R50 (compared to reference mix R0) after 30 and 60 min of water contact, respectively.

### 3.3. Dynamic Elastic Modulus Results

**Figure 10:**
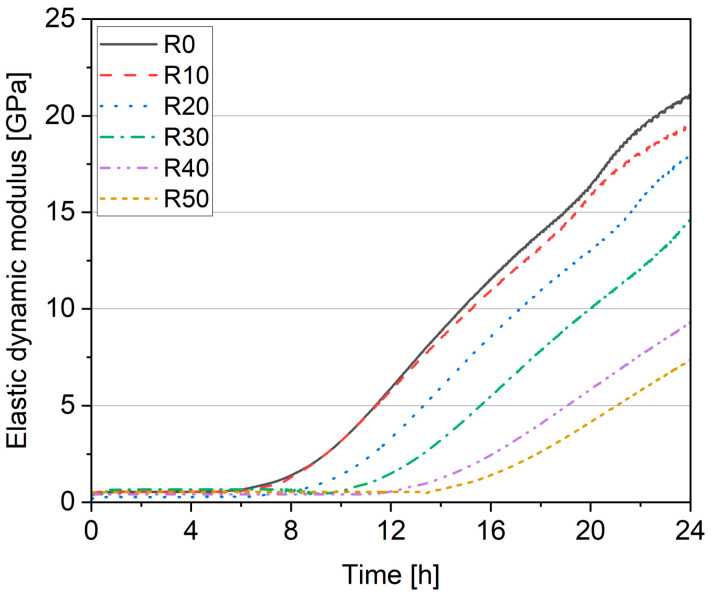
Dynamic elastic modulus development up to 24 h.

Figure 10 presents the development of the dynamic elastic modulus (E_dyn_) of the 3DPC up to 24 h. A gradual decrement in the development of the dynamic elastic modulus can be seen along with an increase in the dosage of cement replacement with fRA. This confirms the delayed hydration process in the 3DPC containing fRA due to the lower cement content and higher effective SP dosage in the binder. At the same time, lower 24-h E_dyn_ was achieved when the fRA was used. The initial build-up of E_dyn_ for reference mix R0 was approximately 5 h 30 min. The increase in the recycled aggregate content delayed the build-up time to approximately 13 h 30 min (R50). The most considerable reduction in E_dyn_ was 64% and was observed for R50 (the mix with the highest content of fRA). Therefore, this test indicates that replacing the cement with fRA leads to a delayed setting time of the 3DPC and E_dyn_.

### 3.4. Hardened Mechanical Properties Results

**Figure 11:**
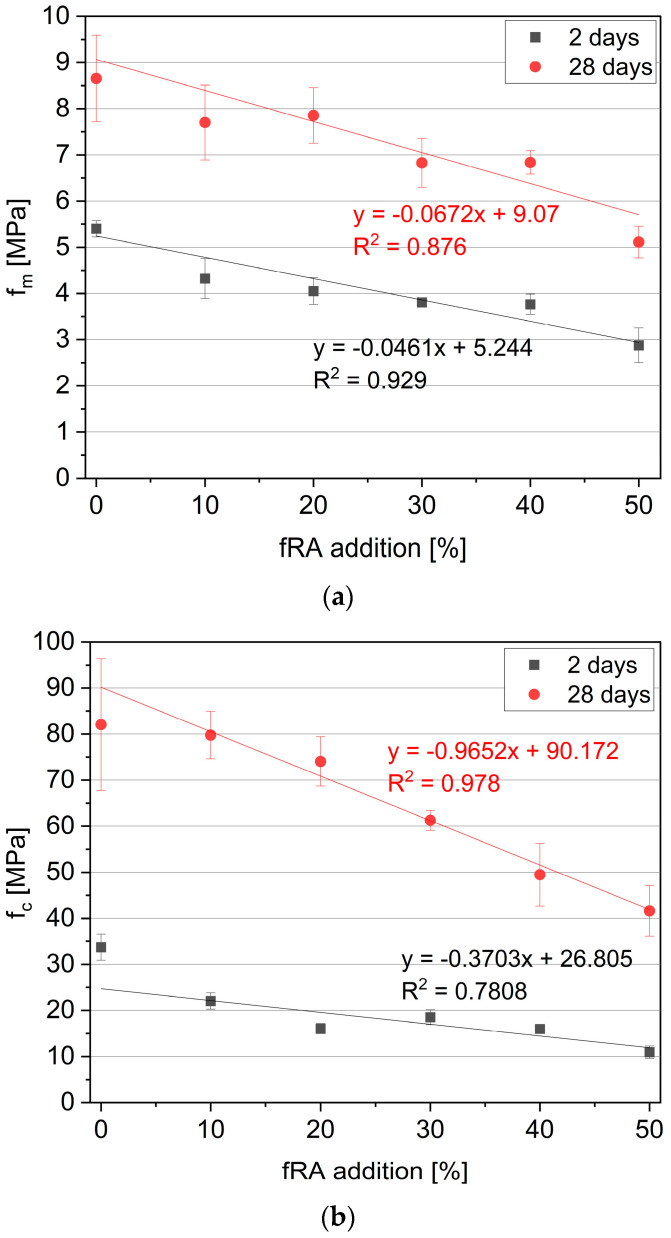
Flexural (**a**) and compressive (**b**) strength results.

The hardened mechanical properties are presented in Figure 11. The data presented in the plot are the mean values of the compressive ($f_{c}$) and flexural ($f_{m}$) strength and their respective CoV. The CoV ranged between 2.38% and 13.04% for flexural strength and between 3.58% and 17.41% for compressive strength. The presented results show that the mechanical properties decreased linearly with the incorporation of fRA. It should be noted that the reduction for flexural strength was up to 46.74%, and compressive strength was up to 67.62% compared to the reference sample (R0). As an outcome mix, R50 exhibited ($f_{c}$ = 41.61 MPa after 28 days, $f_{m}$ = 5.11 MPa after 28 days), which can be considered sufficient for structural applications. However, the replacement of cement with fRA up to 20 vol% resulted in the reduction in the 28-day compressive strength by 9.77% and almost within test scatter, while that for R10 was negligible. However, the 2-day compressive strength of R20 was 52.40% smaller than that of R0. This agrees with the phenomenon described in Section 3.3, as follows: cement replacement with fRA delays hydration due to a lower amount of cement.

### 3.5. Density and Porosity of Tested Mixes

The oven-dry density and open (capillary) porosity values are shown in Table 3. The replacement of cement with fRA resulted in a gradual decrement in oven-dry density and an increase in open porosity. This effect is attributed to the lower density of the fRA compared to the cement and the simultaneous increase in porosity of the specimen. The open porosity of R0 was very low, due to its compact and dense microstructure caused by the high binder content of typical 3DPC mixes (comparable to that of high-performance concrete). The decrease in cement content due to the incorporation of fRA increased the porosity because of the differences in the grading of the cement and fRA particles and because of the higher effective water/cement ratio of the mixes of fRA, along with jamming of the aggregate (being a result of limited paste volume), which resulted in the creation of a porous matrix in the 3DPC mixes containing fRA.

### 3.6. Printing and Buildability Evaluation

The printing was conducted according to the procedure described in Section 2.7. Based on a previous study [35], the nozzle velocity printing was changed to 50 mm/s. For the printing test, the following three mixes were chosen: reference one (R0), a mix with 30 vol% addition of fine recycled aggregate (fRA) (R30), and a mix with 50 vol% addition of fRA (R50).

**Figure 12:**
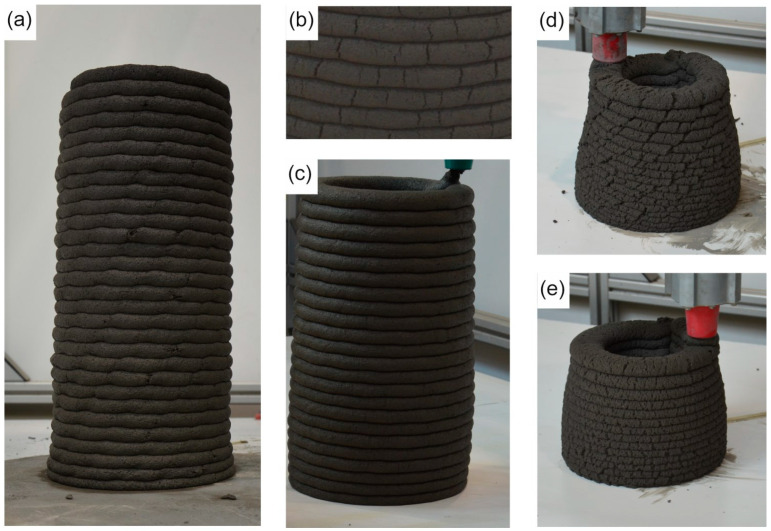
Printing process results: (**a**) specimen R0 during printing; (**b**) specimen R30 surface quality; (**c**) specimen R30 during printing process; (**d**,**e**) specimen R50 during printing process.

Figure 12 shows some photos taken during the printing process. For reference, sample R0 could print a column with a height of 30 layers ± 1 layer (height of 42 cm ± 1.5 cm)—Figure 12a. The column does not have any noticeable deformation during the printing process. In this case, the aim of the buildability test was achieved. For this study, the columns for the R30 and R50 mixes were also printed. For mix R30, the column collapsed after 23 ± 2 layers (height of 32 cm ± 2.5 cm)—example in Figure 12c. In this case, the visible surface defects occurred during the printing process only before the collapse (Figure 12b); therefore, it should be noted that the plastic collapse effect is visible for this kind of mix. Mix R50 exhibited a very low surface quality during the printing test (Figure 12d,e), and the final results allowed printing between 14 and 17 layers in total (height of 21–25 cm). All of the tested samples indicated plastic collapse of the mix (see two examples in Figure 12d,e).

To sum up, an increased fRA content in the mix decreases the allowable print height of columns. It is a consequence of the low green strength observed in the fresh properties tests, caused by decreased thixotropy and buildability due to fewer fines (cement) in the mix. This problem is further discussed in the Discussion section. It should be noted that the increased fRA content led to decreased surface quality, as follows: R0 had only minor surface defects; R30 indicated numerous surface defects, especially visible before collapse; and R50 indicated a very low surface quality. An additional description of the mentioned phenomena is presented in the Section 4.

### 3.7. Life Cycle Assessment

Table 4 shows the environmental impacts (evaluated through the global warming potential) and costs associated with each raw material, along with the respective references [36,37] for the LCA information module A1 (raw material production).

The data for the natural aggregate were sourced from Ecoinvent 3.8 [29] and obtained from the processes “Sand 0/2 mm, wet and dry quarry, production mix, at plant, undried RER S (ELCD)”, while that for water was obtained from the process “Tap Water {RER} market group for|cut-off, U”.

Concerning the fRA, and since (i) recycled aggregates may be produced with very different production processes [18] and (ii) the example presented in this paper assumes a hypothetical production site, to avoid biases, the authors opted to define the global warming potential based on several Environmental Product Declarations concerning the production of recycled aggregates. The following publications were collected [41,42,43,44,45,46] and the mean value of their global warming potential was used. In the case of fRA cost, several factors influence the cost of a recycled aggregate [44], and the costs different from those presented in Table 4 may be more accurate depending on the region.

For the transport distances, we assumed that the 3D printable material production occurred in Szczecin (Poland), which are presented in Table 5. The fRA were assumed to be produced on site, and no transport distance has been attributed to them (as shown at the end of this section): moreover, this has no practical implications on the outcomes of this comparative LCA. Table 5 also presents the costs and global warming potential of transporting raw materials to the factory (information module A2). For the global warming potential shown Table 4, we assumed a 32-ton truck and the process “Transport, freight, lorry > 32 metric ton, euro6 {RER}|market for transport, freight, lorry > 32 metric ton, EURO6|Cut-off” (corresponding to 0.068 kgCO_2_eq./tonkm), retrieved from the Ecoinvent 3.8 database [29]. The transport costs presented in Table 5 were calculated from the mean transport cost of road transportation in Europe for 20-ton cargo in March 2024 [47] (EUR 0.082/tonkm).

The global warming potential and cost of the mixing process were disregarded (information module A3) since they were negligible and well below the cut-off limits—e.g., according to Hajek et al. [40], the mixing process of 1 m^3^ of concrete corresponds to 5.04 kgCO_2_eq.

**Figure 13:**
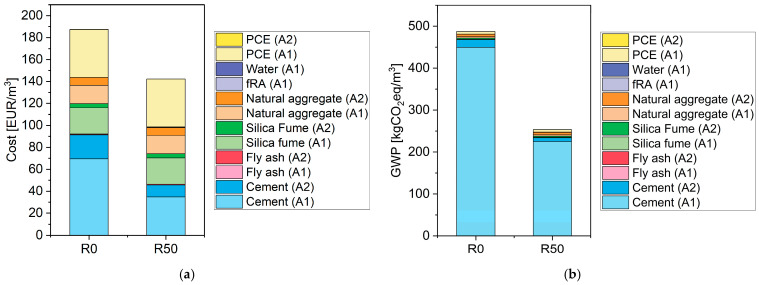
Contribution of each raw material and information module in the global warming potential and cost of R0 and R50: (**a**) cost (the values for Fly ash (A1, A2), Water (A1), PCE (A2), fRA (A1) are very low or equal to 0 (fRA) and hardly visible in the plot); (**b**) global warming potential (the values for Fly ash (A1, A2), Silica Fume (A1), water (A1) and fRA (A1) are very low or equal to 0 (fRA, Fly ash (A1)) and hardly visible in the plot)

Table 6 shows the cost and global warming potential of all mixes. It can be immediately understood that the replacement of cement with fRA results in very relevant decreases in both. Since 49% of the cost and 96% of the global warming potential of R0 is due to cement (Figure 13), and because the impacts and cost of fRA are minimal, it has been found that the relative decrease in cost and global warming potential is almost the same as the replacement ratio of cement with fRA (e.g., for 50% replacement of cement, the cost decreases by 25% and the global warming potential decreases by 48%). These results are expected from the literature. Notwithstanding the scarce number of publications on the global warming potential of mortars for 3D printing, LCA made on concrete shows that cement is responsible for 75% to 90% of the global warming potential of concrete [48]. Since mortars for 3D printing have a higher cement content than that of concrete, the relative importance of cement will increase (as found in this paper).

These results show that using fRA as a replacement for cement greatly contributes to reducing the global warming potential (Table 7), while the cost decreases. Due to the technical concerns put forward in Section 3.1, Section 3.2, Section 3.3, Section 3.4, Section 3.5 and Section 3.6, the authors highlight that the strategy proposed (replacing cement with fRA) should be focused on small replacement ratios (10% or 20%), which have the potential to decrease the global warming potential by 10% to 29% and decrease the cost by up to 10%.

**Figure 14:**
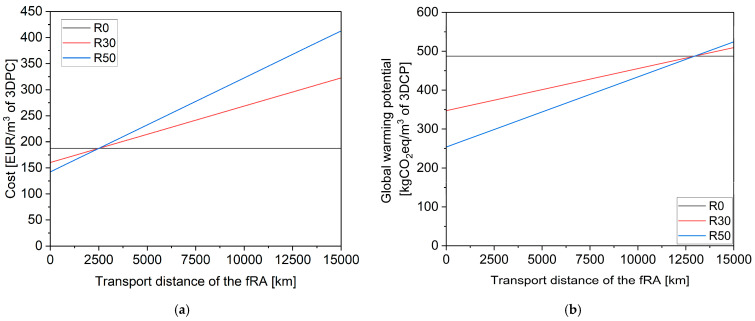
Sensitivity analysis on transport distance: (**a**) cost; (**b**) global warming potential.

Since these data assumed a negligible transport distance from the recycled aggregate producer to the factory, a sensitivity analysis on transport distances was carried out and presented for global warming potential and cost in Figure 14. As seen in the figure and in another publication (in which recycled aggregates replaced natural aggregates in concrete production) [49], the global warming potential of the mortars with fRA equals that of those of the reference for a more considerable transport distance (13,000 km) than that required to equal cost (2500 km). However, as expected, since, in this paper, the fRA replaced cement, it has been found that the extent of the decrease in global warming potential and cost is so large that unrealistically high transport distances are a condition for the replacement of cement with fRA, which is unjustified.

## 4. Discussion

The paper addresses a critical issue related to the development of sustainable 3D concrete printing technology in terms of the reduction in cement content in 3D printed mixes. This study evaluated the potential strategy of substituting cement with fine recycled aggregate produced from recycled 3D printed structures.

The slump flow of the designed mixes after 15 min of water contact aligns with findings from other studies in the field (i.e., [32,33,34]). However, after 30 min of water contact, the mean diameter of the flow for mixes R40 and R50 falls below the printability region, while, for R30, the printability window is shortened. This phenomenon is attributed to the high water absorption rate of the used aggregate; nonetheless, this situation hinders the possibility of printing. Therefore, based only on the slump flow, it can be concluded that replacing cement with fine recycled aggregates (fRA) without modifying the mixture design with chemical admixtures is reasonable for a maximum replacement rate of 20% by volume (mixes R10 and R20).

The evaluation of the green strength and buildability during the printing process indicates that the results are sufficient for the printing process. Firstly, it should be noted that the green strength obtained for materials modified with the fine recycled aggregate (fRA) ranges between 5.27 kPa and 11.09 kPa after 30 min and between 6.26 kPa and 14.73 kPa for specimens tested for 60 min. These values are in line with those presented in other studies [13,15,30,32,50]. Chosen research teams that focused on ordinary mixes without recycled aggregate incorporation obtained the following results after 30 min: Casagrande et al. [32] reported values of between 4.30 kPa and 26.04 kPa, Wolfs et al. [50] reported about 10.5 kPa, Panda et al. [51] reported about 10.65 kPa, and Skibicki et al. [30] reported about 9.02 kPa. The analyses of these studies prove that the obtained results are within the boundaries that demonstrate printing potential. Other studies dedicated to the incorporation of recycled aggregates present inconclusive results. For example, Ding et al. [15] indicate an increase in green strength after incorporating recycled sand, from 10.68 to 12.23 kPa, while Skibicki et al. [13] prove that the addition of recycled aggregate leads to a decrease in green properties (from about 14 kPa to 3.8 kPa). Based on the presented examples, (i) in both studies, the recycled aggregate was used as a substitution for a natural aggregate, while, in the presented paper, the authors substituted the binder; and (ii) the obtained values in the presented papers [13,15] are comparable to the values obtained in this current study.

**Figure 15:**
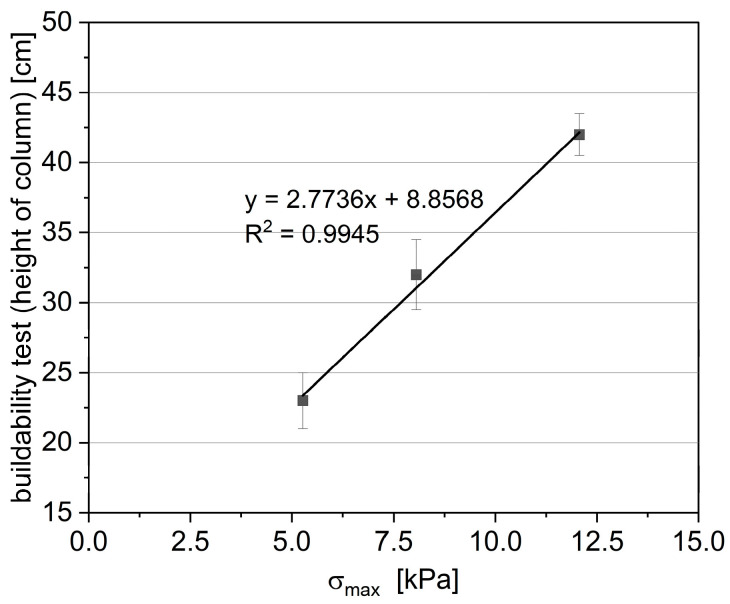
The correlation between green strength $\sigma_{max}$ and printing test results.

A buildability test was performed to assess the printing potential for real-scale verification. It should be noted that the results from the printing test show a linear correlation with the green strength results. Figure 15 illustrates a comparison between the height of the printed structure and the green strength results (measured after 30 min of water contact). These results align with those presented in Figure 7, which show a decrease in static yield stress by adding the fine recycled aggregate (fRA). The samples with an increasing recycled aggregate content and decreasing cement content exhibit a noticeable decrease in the static yield stress growth. However, due to noticeable alterations in the rheological properties of mixes containing fRA, the quality of the material strongly tends to decrease when the fRA content exceeds 20 vol%. Therefore, certain modifications of mix designs and printing process (i.e., slower printing speed) might be required for mixes R30–R50 to ensure the proper quality of printed structures.

The setting time of the mix was evaluated based on the dynamic elastic modulus. The results showed a rapid increase in mechanical properties (initial build-up of E_dyn_) starting after about 5 h and 30 min for mixes R0 and R10, which could be assumed as the final setting time. This time aligns with the time presented in [52] (5.33 h) and is slightly lower than the time presented in this study [53] (7 h and 10 min). For mixes R20–R50, the rapid increase in mechanical properties is delayed (up to 13 h and 30 min for the R50 mix). This phenomenon is related to the increase in the amount of recycled aggregate in the mix, consistent with studies [54,55].

The open porosity analysis revealed a substantial increase in open porosity, due to the reduction in cement content, rising from 2.84% (R0) to 11.36% (R50). This trend of increased porosity due to the mentioned substitution aligns with findings presented in the literature [56,57,58]. However, it is essential to note that different papers have reported varying changes in the porosity for similar substitutions. For example, Guo et al. in 2013 [56] demonstrated that the porosity increased from 12% to 14% for mixes with a 50% substitution of recycled aggregate (only aggregate substitution, the mix with a cement content of 426 kg/m^3^). Muda et al. [58] showed an increase in the porosity from 37% to 43% with a 50% substitution of aggregate (only aggregate substitution, the mix with a cement content equal to 350 kg/m^3^). Anastasiou et al., in 2013 [57] substituted the cement content with recycled aggregate up to 50% and observed an increase in the porosity from 13% to 21% (the cement content in the reference mix was equal to 350 kg/m^3^). It is worth noting that Guo et al. [56] and Muda et al. [58] only substituted the aggregate with recycled material, while Anastasiou et al. [57] replaced the cement (up to 50%) with recycled aggregate. Nevertheless, both sets of studies [56,57,58] demonstrate a significant increase in porosity.

**Figure 16:**
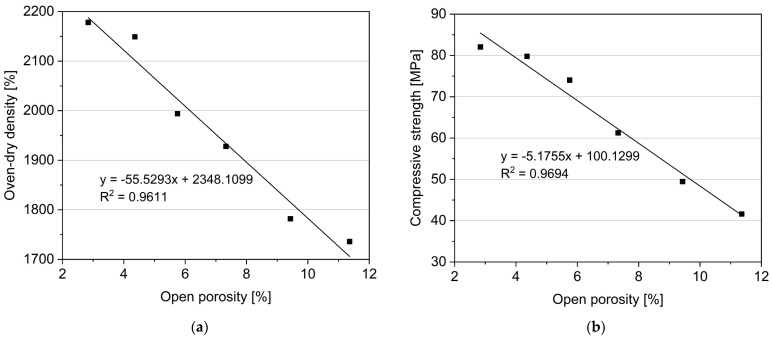
Oven-dry density versus open porosity (**a**) and 28-days compressive strength vs. open porosity (**b**).

The correlation between oven-dry density and open porosity was established (Figure 16), confirming an approximately linear relationship. Similar observations were made for compressive strength and open porosity, affirming the state-of-the-art understanding that porosity has a linear relationship with the compressive strength of composites. From this perspective, replacing cement with fine recycled aggregates (fRA) up to 20% by volume seems to be the most reasonable, as the mechanical strength loss was found to be marginal for mixes R10 and R20. In contrast, the higher replacement rates resulted in noticeable strength deterioration. However, considering that 3D printed concrete mixes are generally regarded as high-performance materials, the R50 specimen exhibited a compressive strength of over 40 MPa, indicating a satisfactory mechanical performance to be considered as a structural material [30,53,59].

## 5. Conclusions

This paper concerned the development of sustainable mortars for 3D printing, aiming at maximum environmental efficiency. In order to achieve this, partial replacements of cement with fine high-quality recycled aggregates produced from 3D printed concrete rejects were attempted. This paper concerns an analysis of the raw materials; mortar development, including fresh and hardened state testing; life cycle assessment to determine the life cycle costs and environmental impacts; and printing runs to evaluate the suitability of the mortars for 3D printing. The following was found:

- It is possible to successfully print mixes with up to 50% cement replacement using fine recycled aggregates (fRA) obtained from waste 3D printed concrete elements. However, specimens with 50% cement replacement have some technical drawbacks (see points below).
- Adding fRA instead of cement increased the aggregate-to-binder ratio (a/b) from a/b = 1.58 to a/b = 2.86. This phenomenon resulted in the deterioration of the fresh properties of the mix. As the a/b ratio increased, the green strength, static yield stress, and buildability decreased.
- This research demonstrates a correlation between the green strength determined by the UUCT test, the buildability during printing, and the static yield stress in the mixes containing fRA. As the static UUCT increased, it became possible to print taller structures, indicating a linear relationship between these properties. The green strength results from the UUCT testing, which are directly linked to buildability during printing, aligned with these findings. The tests indicated that incorporating fRA reduced the green strength by up to 71.9% (for the R50 mix). This correlation is also supported by the static yield stress measured in rheometric research.
- The dynamic elastic modulus test indicated that the hardening process was delayed in the mixes with a high fRA content. This phenomenon was also confirmed by a significant reduction in the mechanical properties observed after two days of water contact (up to a 67.62% reduction compared to the reference mix).
- Increasing the addition of fRA content led to a decrease in the quality of the mix, resulting in visible defects in the samples.
- The open porosity of the mix increased with the addition of fRA, from 2.84% (R0) to 11.36% (R50). This research shows a linear relationship between open porosity and mechanical properties.
- Incorporating fRA significantly reduced the hardened mechanical properties, particularly during the initial curing stages. However, by the 28th day, the R50 mix showed a compressive and flexural strength reduction of up to 49.3% and 41%, respectively. Nevertheless, the R50 mix still demonstrated relatively high mechanical properties ($f_{c}$ = 41.61 MPa after 28 days and $f_{m}$ = 5.11 MPa after 28 days), which are sufficient for the structural application of the designed materials.
- The LCA analysis showed that reducing the cement content and increasing fRA addition resulted in a significant reduction in mix cost (up to 24%) and a substantial decrease in CO_2_ emissions (up to 48%). It is worth noting that even in mixes with low fRA content, where there was a limited reduction in green strength (replacement ratios of cement with fRA of up to 30%), the environmental impact of the mix was very promising. For instance, in the R30 mix, the CO_2_ emissions were reduced by 29% and the cost decreased by 14%.

The research presented in the paper demonstrates that replacing cement with fRA is a promising idea. Although incorporating fRA reduces the green properties of the 3DPC, this approach aligns with sustainable development goals such as reducing CO_2_ emissions and preserving natural aggregates. Moreover, using recycled aggregates reduces the overall material costs. However, further efforts are required to optimize or introduce chemical additives for improving buildability, green strength, and hardened properties.

## Figures and Tables

**Table 1:** Properties of natural sand and fine recycled 3DPC aggregates.

Aggregate Type	Loose Bulk Density	Relative Particle Density	Saturated Surface Dry Density	Oven-Dry Density	24 h Water Absorption
	kg/dm^3^	kg/dm^3^	kg/dm^3^	kg/dm^3^	%
River sand	1.65	2.65	2.63	2.62	0.4
fRA	1.50	2.40	2.21	2.08	6.4

**Table 2:** Mixture design of 3DPC (kg/m^3^).

Mix	CEM I 42,5 R	Fly Ash	Silica Fume	Natural Aggregate	fRA	Water	SP	a/b Ratio	w/b Ratio
	kg/m^3^	kg/m^3^	kg/m^3^	kg/m^3^	kg/m^3^	kg/m^3^	kg/m^3^	-	-
R0	560	160	80	1266	0	225	2.2	1.58	0.28
R10	504	160	80	1266	44	225	2.2	1.76	0.30
R20	448	160	80	1266	88	225	2.2	1.97	0.33
R30	392	160	80	1266	132	225	2.2	2.21	0.36
R40	336	160	80	1266	176	225	2.2	2.50	0.39
R50	280	160	80	1266	220	225	2.2	2.86	0.43

**Table 3:** Oven-dry density and open porosity of 3DPC.

Mix	Oven-Dry Density	CoV	Open Porosity	CoV
	%	%	%	%
R0	2178	1.0	2.84	9.1
R10	2149	3.8	4.36	1.2
R20	1994	0.8	5.75	4.8
R30	1928	1.1	7.33	5.1
R40	1782	0.8	9.43	1.4
R50	1736	1.0	11.36	5.9

**Table 4:** Costs and global warming potential per ton of raw material production. Information module A1.

Raw Material		Cement	Coal Fly Ash	Silica Fume	Natural Aggregate	PCE	fRA	Water
Cost	EUR/ton	124	0.22	299	13.2	19823	2.0	0.31
	Reference	[38]	[38]	[38]	[38]	[38]	[39]	[38]
Global warming potential	kgCO_2_eq./ton	803	0.010	1.22	2.44	2732	1.14	0.246
	Reference	[36]	[37]	[40]	[29]	[40]	See below	[29]

**Table 5:** Cost and global warming potential of transport from raw material production to the mixing and printing location. Information module A2.

Raw Material	Cement	Fly Ash	Silica Fume	Natural Aggregate	PCE	fRA	Water
Distance (km)	480	55	570	70	791	0	0
Location	Chorula	Dolna Odra	Warszawa	Bielinek	Stuttgart	Szczecin	Szczecin
Cost (EUR/ton)	39.12	4.48	46.46	5.71	64.5	0	0
Global warming potential (kgCO_2_eq./ton)	32.68	3.74	38.81	4.77	53.86	0	0

**Table 6:** Cost and global warming potential of each mix.

Mix	Cost (EUR/m^3^)			Global Warming Potential (kgCO_2_eq./m^3^)		
	A1	A2	A1 + A2	A1	A2	A1 + A2
R0	153.8	33.7	187.5	459.1	28.2	487.3
R10	146.9	31.5	178.5	414.2	26.3	440.5
R20	140.1	29.3	169.4	369.3	24.5	393.8
R30	133.2	27.1	160.4	324.4	22.7	347.1
R40	126.4	24.9	151.3	279.5	20.8	300.3
R50	119.5	22.8	142.3	234.5	19.0	253.6

**Table 7:** Relative decrease in cost and global warming potential due to cement replacement with fRA.

Mix	Cost		Global Warming Potential	
	EUR/m^3^	Relative Decrease to R0	kgCO_2_eq./m^3^	Relative Decrease to R0
R0	187.5	-	487.3	-
R10	178.5	5%	440.5	10%
R20	169.4	10%	393.8	19%
R30	160.4	14%	347.1	29%
R40	151.3	19%	300.3	38%
R50	142.3	24%	253.6	48%

## Data Availability

The raw data supporting the conclusions of this article will be made available by the authors on request.
